# Is Oxytocin Induction at Labor a Risk Factor for Developmental Hip Dysplasia?

**DOI:** 10.3390/jcm13195724

**Published:** 2024-09-26

**Authors:** Lütfiye İdil Emral, Ersin Taşkın, Aysun Albayrak, Memnune Arslan, Demet Soylu

**Affiliations:** 1 Department of Pediatrics, Düziçi State Hospital, 80600 Osmaniye, Turkey; dr.aysuny@gmail.com; 2 Department of Orthopaedics and Traumatology, Düziçi State Hospital, 80600 Osmaniye, Turkey; ersintaskin1989@gmail.com; 3 Department of Pediatrics, Osmaniye State Hospital, 80000 Osmaniye, Turkey; dr.arslanmemnune@gmail.com; 4 Department of Pediatrics, Lokman Hekim University Hospital, 06510 Ankara, Turkey; drdemetsoylu@gmail.com

**Keywords:** developmental hip dysplasia, oxytocin induction, labor induction, hip ultrasonography

## Abstract

**Background:** Developmental hip dysplasia is a common condition with preventable causes, and its etiology is still not fully elucidated. In our study, we aimed to examine the use of synthetic oxytocin during childbirth as a potential risk factor for developmental hip dysplasia. **Methods:** This study involved comprehensive hip examinations on postnatal days 0, 14, 30, and 60, as well as hip ultrasonography results at 6-8 weeks. We specifically focused on healthy girls born with vaginal deliveries, comparing those who were applied with a low-dose oxytocin induction protocol (Group 2) and those who had vaginal deliveries without induction (Group 1). **Results:** When the examination findings were compared with the hip ultrasonography findings (Type 2a was detected in the left hip of one patient (6.3%) in Group 1 and in the right hip of two patients (11.8%) in Group 2), it was seen that oxytocin induction did not cause a risk for developmental hip dysplasia. The oxytocin induction rate was higher in newborns weighing more than 3400 g (*p* = 0.04). **Conclusions:** A low-dose oxytocin protocol applied at birth has not been shown to harm the hip joint in the neonatal period and on ultrasonographic α and β angle measurements applied at 6-8 weeks. However, our study also highlights the need for new studies investigating oxytocin peripheral receptors and their effects, underscoring the importance of our findings in guiding future research in this area.

## 1. Introduction

Developmental hip dysplasia (DDH) describes abnormal conditions ranging from instability to dislocation that begin in the prenatal stage and continue postnatal. The frequency of DDH, the most common musculoskeletal disorder in infants, varies between 0.06 and 76.1 per 1000 according to different races [1]. The incidence of DDH varies according to the geographic and ethnic origin. Although it is thought that gender, applied screening programs, and measurement techniques may also affect the prevalence, there is not enough evidence [2]. DDH is also seen in children without accompanying or underlying diseases. Its exact etiology is still unclear. In managing developmental hip dysplasia, it is essential to determine risk factors, complete a physical examination, and support the diagnosis with imaging methods [3,4].

Examination findings (especially the Barlow maneuver, which tests for hip instability, and the Ortolani maneuver, which detects hip dislocation) are essential in diagnosing DDH in the neonatal period. The Barlow and Ortolani maneuvers are widely accepted screening methods for neonatal hip examination. However, they can be misleading, yielding false-negative results, especially in cases of bilateral hip dislocation [5]. In later months (>3 months), the Galleazi sign, which indicates hip asymmetry, is also meaningful.

Ultrasonography is the gold-standard diagnosis method postnatally in the first six months. Ultrasonographic screening programs are recommended to be routinely implemented, as examination findings can be potentially misleading [5,6]. On the Graf scale, hips are grouped according to their morphological characteristics (the bony and cartilage roof between the femur and acetabulum) and the α and β angles: Type 1 (normal, 1a: beta angle < 55, 1b: beta angle > 55); type 2a (immature); types 2b and 2c (minor dysplasia); and Graf type 2d and more advanced stages (major dysplasia requiring treatment) [7,8].

When diagnosed early, DDH can be treated with simple applications (such as a Pavlik harness). However, in the later period, treatment becomes difficult (such as closed reduction, open reduction, femoral and pelvic osteotomy surgeries, and radiological follow-ups) and can cause permanent morbidity. Understanding bilateral hip dislocation with a simple examination is more challenging since the findings will be symmetrical. For these reasons, while discussions on establishing a routine screening protocol continue, ultrasonographic screening is widely used today [9].

In addition to the genetic predisposition and ethnic origin, the risk factors that are effective in the developmental process include breech presentation, swaddling, oligohydramnios, and being the first-born child. Changes in the mother’s hormonal system during pregnancy (hypothyroidism, phenylketonuria, and progesterone exposure in the first trimester), exposures during this period (amniocentesis), and the mother’s habits (smoking) may also be risk factors [9,10].

DDH is a common disorder, but its etiology has not been fully elucidated. The fact that the disease is also seen in children without proven and accepted risk factors plays an important role in the continuing etiology research. In recent years, studies aiming to elucidate the etiology of DDH have focused on genetics, molecular mechanisms, and hormonal theories [11,12,13,14]. The newborn’s hip is known to be unstable in the early period and develops and becomes stable over the weeks [4]. This situation shows the importance of researching the hormones whose levels change at birth.

Hormonal theory constitutes a critical dimension to investigate in the etiology of DDH. Hormones, especially estrogen and progesterone, are investigated in the mother and the infant [9,10]. The effect of estrogen on peripheral receptors has been investigated, and it has been reported that the amount of estrogen receptors in ligamentum teres biopsies of patients with DDH has been increased compared to both joint hip capsule biopsies and control group ligamentum teres biopsies and, therefore, may play a role in the etiology of DDH [15]. It has been reported that estrogen and vitamin D receptor gene polymorphisms may also have an effect on the development and severity of DDH, that Taq I vitamin D receptor polymorphisms may be associated with an abnormal acetabular morphology, and that the Xba I estrogen receptor XX genotype may cause an increase in the risk of DDH [16]. The thyroid hormone has been investigated for its effects on bone metabolism and muscle development, and it has been reported that maternal hyperthyroidism may have an effect on DDH [17]. It has been reported that relaxin may have an effect on the etiology of DDH by affecting collagen synthesis and connective tissue laxity via estrogen and progesterone [13]. When the hormonal theory is considered, oxytocin may be an essential hormonal factor. We aimed to investigate the oxytocin hormone, which has not been previously discussed in the etiology of DDH, but which we think may be effective.

Oxytocin is an ancient hormone that plays a vital role in human health at the level of emotional regulation, anti-inflammatory, autism, osteoarthritis, etc., with side effects and it still needs research [18]. Although oxytocin, a classic peptide hormone, is primarily produced by the hypothalamus, it is also made in the uterus, placenta, and amnion [19]. Changes in oxytocin levels also regulate neural, cognitive, endocrine, and physical activities. Oxytocin cells are found in peripheral regions and the brain and provide autocrine and paracrine regulation. Synthetic oxytocin administered during birth can cross the placenta and blood-brain barrier, though in meager amounts [20,21]. Oxytocin in the peripheral circulation causes metabolic effects such as lipolysis, increased fatty acid oxidation, brown adipose tissue thermogenesis, bone mineralization, and skeletal muscle regeneration [22]. All diseases and processes associated with oxytocin can be modulated by infection, inflammation, or the modification of oxytocin receptor-mediated signaling [23].

Considering the studies conducted with oxytocin, we aimed to examine oxytocin induction as a risk factor for DDH through two different theories. Oxytocin, a hormone that plays a crucial role in childbirth, is often used to induce labor. Could the increased uterine contractions and pelvic floor muscle activity caused by oxytocin induction have a mechanical effect and hurt the hip joint? This is one of the theories we sought to investigate. The other theory we explored is whether oxytocin, administered during labor, could be a risk factor in induced labor through peripheral oxytocin receptor regulation. By examining these two theories, we aimed to provide a comprehensive understanding of the potential relationship between oxytocin induction during labor and developmental hip dysplasia. These findings could inspire and guide future research in the field of pediatric care and obstetrics, offering new avenues for exploration and discovery.

## 2. Materials and Methods

### 2.1. Study Protocol

This study was conducted at Düziçi State Hospital between 1 March 2023 and 1 March 2024. Healthy female children born vaginally, with or without oxytocin induction, were included in the study. Parents who agreed to participate in the study were informed that the mother and child’s demographic data and examination findings would be used, and their consent was obtained. All mothers were followed up for vital signs and uterine hyperstimulation throughout labor. Fetuses were monitored with cardiotocography. Postnatal hip examination findings of the baby (postnatal days 0, 14, 30, and 60) and hip ultrasonography results performed between 6 and 8 weeks were recorded.

Obstetricians and gynecologists were excluded from the study to ensure randomization and prevent bias. Thus, it was ensured that oxytocin was administered to the mother only due to medical necessity and that there was no bias. The obstetrician determined whether the mother needed or did not receive oxytocin at labor. Whether oxytocin was administered, and if administered, the dosage and protocol, were reported to the researchers in a sealed envelope. After completing the study protocol, it was opened for grouping purposes and evaluated, ensuring the highest level of research integrity and thoroughness.

1. Oxytocin application protocol

A low-dose oxytocin induction protocol was applied to patients. It was learned that oxytocin induction continued until the end of labor, and no reduction in the oxytocin induction rate was required in any patient.

2. Ultrasound screening protocol

Hip ultrasound imaging was performed in the lateral decubitus position, with the hip and knee in semi-flexion. Ultrasonography was applied to all patients using the same device, and all images were obtained using a 7.5 MHz linear probe. In all scans, a morphological evaluation (acetabular–femoral conformation, the shape of the cotyloidal margin, and thickness of the acetabular cartilage) was first performed; then, for quantitative evaluation, the α and β angles were measured, and the hip type was determined according to the Graf scale. 

### 2.2. Sample Size

No formal sample size calculation was performed. The sample size was determined by referencing similar studies [24]. A similar study was conducted to address the effect of oxytocin induction applied at birth on developmental hip dysplasia in newborns (the incidence reported by Sioutis et al. ranges from 0.06 per 1000 live births to 76.1 per 1000 live births). We calculated a sample size of 33, with 80% power and 5% significance [1].

### 2.3. Data Collection

1. Inclusion criteria

Mothers who underwent routine check-ups before and during pregnancy and who did not detect any pathology in their examination findings and laboratory tests were included in the study. Data were recorded for healthy girls born via normal vaginal delivery over a one-year period, whose families accepted their participation in the study. Since ethnic origin and genetic predisposition are essential in the etiology of DDH, only patients of Turkish origin were included in the study; individuals of other nationalities were not included. Newborns with a cephalic presentation during labor were included in the study. Patients who applied the low-dose oxytocin induction protocol were included in the study because it is more suitable for the physiological course of labor and has a lower risk of uterine hyperstimulation and tachysystole [25]. Therefore, it was thought that the high-dose protocol might negatively affect the study data. Mothers who had a single pregnancy, were not diagnosed with oligohydramnios or polyhydramnios, and were not undergoing amniocentesis were included in the study.

2. Exclusion criteria

Boys, preterm newborns, patients with congenital anomalies or genetic diagnoses, and patients diagnosed with a chronic disease were excluded from the study. Breech presentation and others were excluded. Mothers who became pregnant through in vitro fertilization treatment, had multi-fetal pregnancies, or smoked during pregnancy were excluded from the study. This was to maintain a homogenous study population and to eliminate potential confounding factors. Newborns whose labor began with vaginal delivery but were born by cesarean section for various reasons were excluded from the study. This was to ensure that the study focused on the effects of oxytocin induction during vaginal delivery. Infants who did not continue their follow-up, for any reason, did not have hip ultrasonography between 6 and 8 weeks, and patients who were swaddled to include the hips in the 0–2 month period were excluded from the study. This was to ensure that the data collected were complete and reliable. 

### 2.4. Data Preparation and Grouping

When the patients’ follow-up was terminated, their oxytocin information was included in the study. Newborns of mothers who received oxytocin at doses ranging from 0.5 to 6 mU/min (low-dose protocol) were included in the study. In contrast, those who received lower or higher doses were excluded [26]. Patients who did not receive induction during labor were designated as Group 1 (control), and those who received induction due to medical necessity were designated as Group 2 (patient). The examination findings and hip ultrasonography results of the groups were compared, and the effect of oxytocin induction was investigated. The Graf method was used as the basis for hip ultrasonography and typing, and type 2a and advanced stages were accepted as DDH [8]. Patients diagnosed with hip dysplasia due to examinations or ultrasonography were referred to the orthopedics department.

### 2.5. Ethics Committee Approval

The study approval was received from the Adana Training and Research Hospital Clinical Research Ethics Committee. The ethics committee approval decision number is 121/2384. The principles of the Declaration of Helsinki conducted this study.

### 2.6. Statistical Analysis

Calculations were performed using the Statistical Package for Social Sciences (SPSS 22.0, IBM Corp., Armonk, NY, USA) program. The Kolmogrov–Smirnov test was applied to assess whether the variables were normally distributed. Categorical data were compared using the Chi-square test and Fisher’s Exact Test. Independent numerical variables were analyzed using the Student *t*-test. The Pearson test was used to determine the relationships between the study parameters. The significance level was shown as *p* < 0.05.

## 3. Results

Thirty-three patients who met the criteria (eleven patients were excluded from the study for various reasons) were included and continued their follow-up until the end of the study. Newborns who were not administered oxytocin at labor constituted Group 1 (*n* = 16), and newborns whose labor was induced with oxytocin constituted Group 2 (*n* = 17). The mean duration of oxytocin induction was 11.5 ± 4.3 h. The median duration of oxytocin induction was 10.1 h (min: 6.17, max: 18.7 h). The labor durations of the two groups were compared, and no significant difference was found (*p* = 0.28).

No difference was found when the two groups were compared in terms of maternal age (Group 1: 29.1 ± 5.8 years, Group 2: 27.9 ± 7.9 years), gestational age (Group 1: 39.5 ± 0.7 weeks, Group 2: 39.6 ± 0.7 weeks), and infant length (Group 1: 49.2 ± 1.4 cm, Group 2: 49.4 ± 1.2 cm). The birth weight of the infant in Group 2 (3403.5 ± 404.2 g) was higher than in Group 1 (3151.3 ± 262.7 g) (*p* = 0.04).

There were no findings suggesting hip dysplasia in any newborn in the physical examinations performed on postnatal 0 and 14 days. During the follow-up of the patients, no positive findings were found in any of the patients as a result of the Barlow and Ortolani maneuvers. At the 30th day’s examination, pili asymmetry was detected in four infants (25%) in Group 1 and three infants (17.6%) in Group 2. Since pili asymmetry is a finding that can occur in healthy children and other examinations were normal, hip ultrasonography was not deemed necessary in the early period. In Group 2, one of the patients with pili asymmetry was found to have a type 2a hip, but this was not statistically significant (3.03%, *p* > 0.05). At the 60th day’s examination, an abduction limitation was detected in two infants (12.5%) in Group 1 and one infant (5.9%) in Group 2. In Group 2, the patient with limited abduction was diagnosed with a Graf type 2a in her right hip using ultrasonography. In Group 1, one patient with the same limitation was staged with type 1b, while the other had type 2a in their left hips.

In Group 2, type 2a was detected in the right hip of two patients (the oxytocin induction lasted 16 h and 35 min for one patient and 10 h and 10 min for the other), and type 1b was detected in the right hip of two patients (the oxytocin induction lasted 6 h and 22 min for one patient and 15 h and 7 min for the other). In Group 1, type 1b was detected in the left hip of two patients, and type 2a was detected in the left hip of one patient. No bilateral instability or dysplasia was detected in any patient. The hip ultrasonography results are shown in Table 1 according to the Graf stages. Type 2a was detected in the left hip of one infant (6.3%) in Group 1 and in the right hip of two infants (11.8%) in Group 2. A comparison of the ultrasonographic α and β angle measurements of the infants is presented in Table 2. No significant difference was found between the groups’ Graf hip types and ultrasonographic α and β angle measurements.

## 4. Discussion

Developmental hip dysplasia is a preventable health problem. Therefore, the risk factors, diagnosis, and treatment methods that trigger it in the intrauterine and postnatal periods are still being investigated [4].

Pelvic floor muscles exhibit phasic activity with pain and increased intra-abdominal pressure (such as labor), creating strong reflexes and contractions [27]. Synthetic oxytocin administered during labor causes intense and painful contractions, disrupting the physiological contraction reflex and coordination in the pelvic floor muscles. Oxytocin induction increases the intensity and frequency of uterine contractions; therefore, the perception of labor pain is higher. The rate of contraction of the pelvic floor muscles may be higher in women receiving oxytocin due to the induction of pain sensation [28].

It is known that oxytocin and estrogen, especially in women, have an anabolic effect on bone metabolism together with estrogen [29]. Peripheral oxytocin can affect the microstructure of joints and bones by increasing the activity of osteoblasts and chondroblasts [30]. The hormone oxytocin influences the differentiation of human muscle cells by impacting the rate of myoblast fusion. It has varying effects on different muscle groups [31]. Oxytocin is recognized for its effectiveness in skeletal and cardiac muscles, muscle contraction, and thermogenesis [32]. Oxytocin receptors have been shown in the uterosacral ligament, arteries, veins, and nerves [33]. 

Unstable hip types are seen more frequently in ultrasonographic imaging performed before 6–8 weeks, possibly due to the lack of down-regulation of oxytocin receptors in the hip joint. We have not come across any studies on oxytocin and its receptors in the acetabular joint ligaments. In the examination findings and imaging results, no adverse effects of oxytocin were found via possible peripheral receptors (via the acetabular ligaments). However, the ultrasonography results performed within the first 24 h after birth and the data on boys will be necessary.

Our study observed that oxytocin application was more common in newborns weighing > 3400 g. Although the literature reports a tendency to apply a high-dose oxytocin protocol in newborns weighing > 4000 g, this is seen as controversial [34,35].

A low-dose oxytocin induction protocol was applied to the patients included in our study. It was learned that synthetic oxytocin induction was started at 0.5 mU/min and continued with 1–2 mU/min increases at 15–40 min intervals, and the maximum dose applied was 6 mU/min. Our study reflects the results of the low-dose protocol. In the study conducted by Daly et al., it was reported that the synthetic oxytocin rate reached 25–33 mU/min in high-dose protocols [36]. High doses of oxytocin can easily cross the placenta, leading to potential side effects [37]. All data may change in high-dose protocols.

Although the dose-dependent effects of oxytocin on central and peripheral receptors are known, its impact and regulation are still not fully understood [38,39]. The effects and regulation of oxytocin are still not fully understood [40]. Expanding current research and data, expediting animal experiments, and even conducting pharmacokinetic and pharmacodynamic studies personally may also be advantageous. Large and prospective cohort studies examining the effects of oxytocin are also needed [41].

The oxytocin receptor contains genetic variations and undergoes epigenetic mutations. Mutations are thought to be more dynamic in infancy than in adulthood. Interactions in the oxytocin system may also vary depending on the region where the receptor is located and gender [40,41,42]. Studying genetic variations and epigenetic mutations in oxytocin receptors in normal hips, unstable hips, and dysplastic hip babies would be helpful.

Our study, which shares the results of low-dose oxytocin administration, underscores the need for further investigation into high-dose oxytocin administration. Research conducted using ultrasonography and clinical examinations immediately after birth and during follow-up can provide significant insights. Additionally, understanding the molecular mechanism, genetic basis, and variations of the oxytocin receptor in the acetabular capsule and surrounding ligaments of newborns and infants with and without DDH is a crucial area for future research.

## 5. Limitations of the Study

This study was conducted with a limited number of patients. A low-dose oxytocin induction protocol was applied, but it did not provide information on high doses. The patients’ follow-ups for the study were terminated on the 60th postnatal day, and the effect on the later period and walking was not investigated. Although there were a small number of primiparous patients in the study, selecting multiparous patients may provide more accurate results. Ultrasonographic measurements of the α and β angle and Graf typing in the first 24 h after birth may help to clarify the effect in the acute period. We believe that studies conducted only with male patients are also critical.

## 6. Conclusions

The low-dose oxytocin protocol applied at labor has not been shown to negatively affect the hip joint in the neonatal period or the ultrasonographic α and β angle measurements applied at 6-8 weeks. New treatments are needed to investigate oxytocin peripheral receptors and their effects.

## Figures and Tables

**Table 1:** Graf hip types according to ultrasonographic angle measurements of the groups.

*n* (%)	Oxytocin			
	Induced (*n*: 17)		Non-Induced (*n*: 16)	
**Graf Stages**	**Right Hip**	**Left Hip**	**Right Hip**	**Left Hip**
Type 1A	13 (78.4%)	17 (%100.0%)	16 (100.0%)	13 (81.3%)
Type 2A	2 (11.8%)	-	-	1 (6.3%)
Type 1B	2 (11.8%)	-	-	2 (12.5%)
Total	17 (100.0%)		16 (100.0%)	

**Table 2:** Comparison of the ultrasonographic α and β angle measurements of the infants.

Parameter Mean ± SD	Oxytocin		*p*
	Induced (*n*: 17)	Non-Induced (*n*: 16)	
Right Hip α angle	63.7 ± 5.8	62.9 ± 4.0	0.67
Left Hip α angle	64.3 ± 4.5	61.6 ± 3.5	0.69
Right Hip β angle	46.8 ± 8.9	47.9 ± 6.8	0.06
Left Hip β angle	45.9 ± 11.2	48.8 ± 7.1	0.39

## Data Availability

The data presented in this study are available on request from the corresponding author.
